# Elimination of tumor hypoxia by eribulin demonstrated by ^18^F-FMISO hypoxia imaging in human tumor xenograft models

**DOI:** 10.1186/s13550-019-0521-x

**Published:** 2019-06-03

**Authors:** Songji Zhao, Wenwen Yu, Naoyuki Ukon, Chengbo Tan, Ken-ichi Nishijima, Yoichi Shimizu, Kei Higashikawa, Tohru Shiga, Hiroko Yamashita, Nagara Tamaki, Yuji Kuge

**Affiliations:** 1 Department of Tracer Kinetics and Bioanalysis, Graduate School of Medicine, Hokkaido University, Sapporo, Japan; 2 Advanced Clinical Research Center, Fukushima Global Medical Science Center, Fukushima Medical University, 1 Hikariga-oka, Fukushima City, Fukushima 960-1295 Japan; 3 Central Institute of Isotope Science, Hokkaido University, Sapporo, Japan; 4 Department of Integrated Molecular Imaging, Graduate School of Medicine, Hokkaido University, Sapporo, Japan; 5 Faculty of Pharmaceutical Science, Hokkaido University, Sapporo, Japan; 6 Department of Nuclear Medicine, Graduate School of Medicine, Hokkaido University, Sapporo, Japan; 7 Department of Breast Surgery, Graduate School of Medicine, Hokkaido University, Sapporo, Japan

**Keywords:** Eribulin, Tumor hypoxia, ^18^F-FMISO PET imaging, Remodeling of tumor vasculature, Human tumor xenograft model

## Abstract

**Background:**

Eribulin, an inhibitor of microtubule dynamics, shows antitumor potency against a variety of solid cancers through its antivascular activity and remodeling of tumor vasculature. ^18^F-Fluoromisonidazole (^18^F-FMISO) is the most widely used PET probe for imaging tumor hypoxia. In this study, we utilized ^18^F-FMISO to clarify the effects of eribulin on the tumor hypoxic condition in comparison with histological findings.

**Material and methods:**

Mice bearing a human cancer cell xenograft were intraperitoneally administered a single dose of eribulin (0.3 or 1.0 mg/kg) or saline. Three days after the treatment, mice were injected with ^18^F-FMISO and pimonidazole (hypoxia marker for immunohistochemistry), and intertumoral ^18^F-FMISO accumulation levels and histological characteristics were determined. PET/CT was performed pre- and post-treatment with eribulin (0.3 mg/kg, i.p.).

**Results:**

The ^18^F-FMISO accumulation levels and percent pimonidazole-positive hypoxic area were significantly lower, whereas the number of microvessels was higher in the tumors treated with eribulin. The PET/CT confirmed that ^18^F-FMISO distribution in the tumor was decreased after the eribulin treatment.

**Conclusions:**

Using ^18^F-FMISO, we demonstrated the elimination of the tumor hypoxic condition by eribulin treatment, concomitantly with the increase in microvessel density. These findings indicate that PET imaging using ^18^F-FMISO may provide the possibility to detect the early treatment response in clinical patients undergoing eribulin treatment.

## Background

Hypoxia, a common characteristic in solid tumors, appears in regions where tumor cells rapidly outgrow the blood supply to form a significantly low-oxygen-concentration environment. Hypoxic microenvironments influence many biological factors that are strongly associated with tumor proliferation, malignant progression, metastasis, chemo-/radio resistance, and poor treatment outcome [1, 2]. With worsening hypoxia and increasing interstitial fluid pressure, pathological angiogenesis gradually develops in tumors. Thus hypoxia can stimulate vascularization; however, the tumor microvasculature shows high abnormality and weak functionality and often fails to rectify oxygen deficiency or improve perfusion and delivery of chemotherapeutics deep into the regions of a tumor [3, 4].

Eribulin mesylate (eribulin), a non-taxane synthetic inhibitor of microtubule dynamics, shows an antitumor capacity against a wide variety of solid cancers and is an FDA-approved medicament for breast cancer (November 2010) and liposarcoma (January 2016) [5–7]. Unlike other microtubule inhibitors, eribulin has a unique mode of action of sequestering tubulin into non-functioning aggregation to prevent normal mitotic spindle formation, leading to irreversible mitotic blockage and apoptosis-inducing activation [8]. Eribulin can also reverse the process from epithelial-mesenchymal transition (EMT) to mesenchymal-epithelial transition (MET) by increasing the tumor expression level of epithelial markers, promoting a phenotypic shift from EMT to MET states, decreasing cellular migration and invasiveness capacities, and pretreating tumor cells to decrease their capacity to colonize [9].

In addition, eribulin has potential use for targeting abnormal tumor vessels, remodeling vasculature through its novel antivascular (antiangiogenesis and vascular-disrupting) activity and improving tumor perfusion to enhance the delivery of chemotherapeutic drugs and reduced tumor core hypoxia to increase the sensitivity to radiotherapy [10]. Recent studies have also identified that eribulin has combination activity with multiple agents from different classes in several human cancer models, including breast, non-small cell lung cancer, ovarian, and melanoma [11]. Although these findings suggest the potential of eribulin to improve tumor hypoxic condition, it remains to be clarified. On the other hand, positron emission tomography (PET) using ^18^F-fluoromisonidazole (^18^F-FMISO) has been recognized as a non-invasive method of imaging hypoxic tumors. It has been used for several types of tumor, including brain, head and neck, lung, and kidney tumors [12–15]. As a 2-nitroimidazole compound, ^18^F-FMISO enters into cells by passive diffusion, where it is reduced by nitroreductase enzymes to be trapped in cells and forms reactive oxygen radicals with reduced tissue oxygen partial pressure (pO_2_) [16]. The low pO_2_ prevents the reoxidation of ^18^F-FMISO metabolites, resulting in ^18^F-FMISO accumulation covalent binding to macromolecular cellular components and glutathione conjugation following the reduction of its nitro group [17, 18]. Accordingly, PET using ^18^F-FMISO can visualize hypoxic tumors. On the basis of these previous findings, we utilized ^18^F-FMISO to clarify the effects of eribulin on the tumor hypoxic condition in comparison with histological findings in mice bearing a human solid cancer cell xenograft.

## Materials and methods

### Radiopharmaceutical and reagent

^18^F-FMISO was obtained from the Hokkaido University Hospital Cyclotron Facility (Sapporo, Japan), which was synthesized as previously described [19–21]. Eribulin mesylate (E7389, Halaven) was kindly provided by Eisai Co., Ltd (Tokyo, Japan).

### Animals

Twenty-three female BALB/c athymic nude mice (9 weeks old; mean body weight, 23.8 ± 1.4 g) were purchased from Japan SLC, Inc. (Hamamatsu, Japan). All mice were housed in a 12-h light/dark cycle with the room temperature maintained at 23–25 °C and the relative humidity maintained 45–60%. Food and water were provided ad libitum and met all the criteria of the Association for Assessment and Accreditation of Laboratory Animal Care (AAALAC) International. All experiments and animal surgical procedures were approved by the Laboratory Animal Care and Use Committee of Hokkaido University (approval number 13-0057) and performed in accordance with the Guidelines for Animal Experiments at the Graduate School of Medicine, Hokkaido University.

### Human tumor xenograft models

**Fig. 1:**
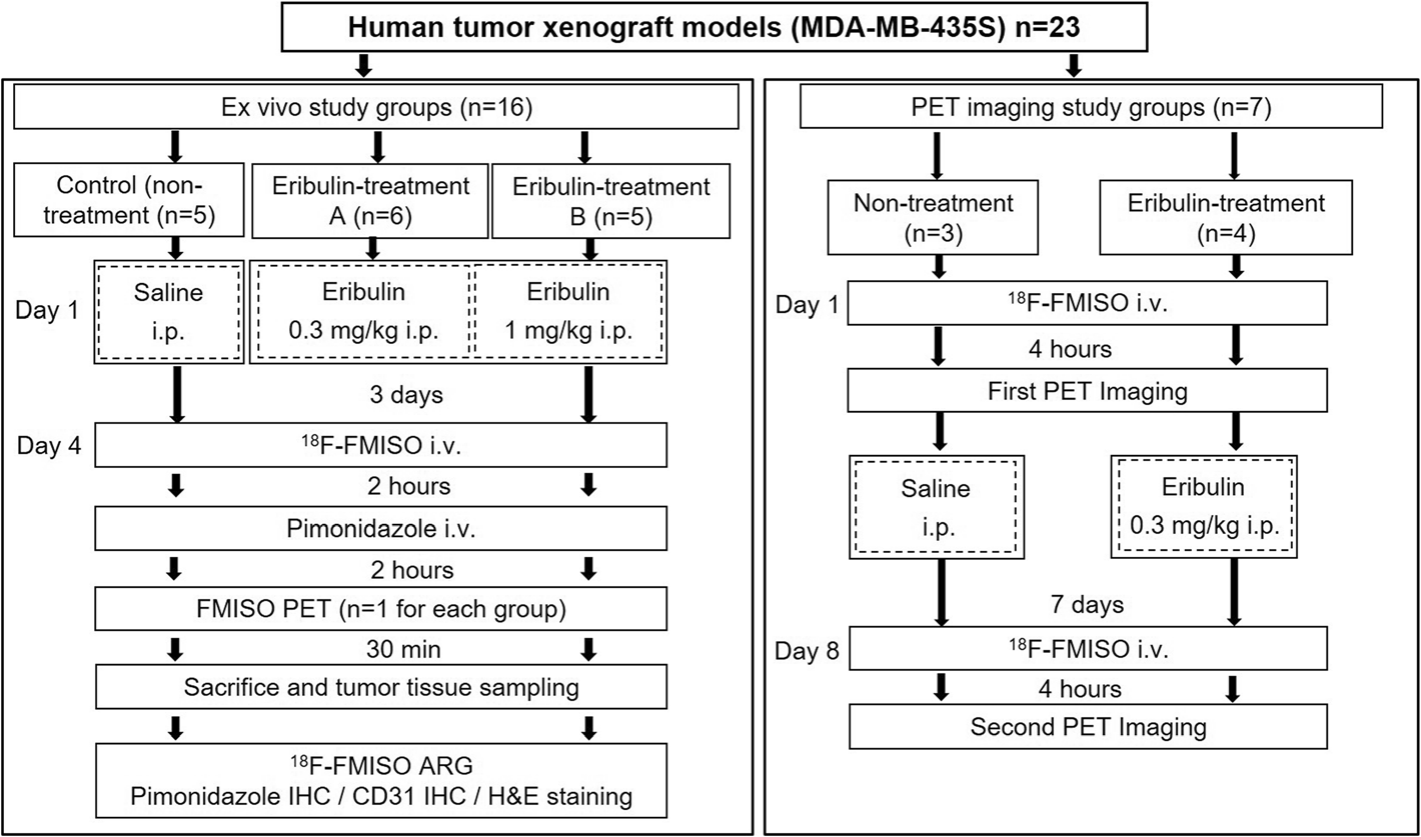
Experimental protocol of this study. The left panel shows the ex vivo study with control (non-treatment) and two eribulin treatment (A, 0.3 and B, 1.0 mg/kg) groups. The right panel shows PET imaging study with control (non-treatment) and eribulin treatment (0.3 mg/kg) groups

The human tumor xenograft models were generated following the experimental protocol shown in Fig. 1. The human MDA-MB-435S cell line, which was originally derived from pleural effusion of a ductal carcinoma patient but was recategorized as a melanoma cell line based on genetic background, was purchased from the American Type Culture Collection (Manassas, VA, USA). Animals were initially anesthetized with 3–4% isoflurane in air and maintained via spontaneous ventilation with 2% isoflurane in air. An MDA-MB-435S cell suspension (1 × 10^7^ cells) diluted of 0.1 ml PBS (-) was subcutaneously inoculated into the right mammary fat pad of each mouse using a 26 G syringe. When the tumor volumes reached 200–400 mm^3^, mice were randomly assigned to the ex vivo (*n* = 16) and PET imaging (*n* = 7) study groups (Fig. 1).

### Ex vivo study

Mice in the ex vivo study group were intraperitoneally administered a single dose of eribulin (0.3 mg/kg body weight, *n* = 6 or 1.0 mg/kg body weight, *n* = 5) or saline (control, *n* = 5) on day 1 (Fig. 1). Three days after eribulin treatment (day 4), the animals were anesthetized and injected with 18.5 MBq of ^18^F-FMISO and 60 mg of pimonidazole per kilogram body weight (a hypoxia marker for immunohistochemistry) into the tail vein 4 h 30 min and 2 h 30 min before sacrifice, respectively. Their body weight and tumor size were measured on days 1 and 4, respectively. Tumor volume was calculated using the following formula: π/6 × (largest diameter) × (smallest diameter)^2^. One mouse from each group was imaged by ^18^F-FMISO PET before sacrifice (detailed PET imaging methods are described in the “^18^F-FMISO PET/CT studies” section). After sacrifice, tumors and other organs were excised. The tumors were quickly sectioned into two blocks. One block was embedded in Tissue-Tek medium and frozen in isopentane/dry ice for autoradiography (ARG) and immunohistochemical analysis. The remaining block was used for the following tissue counting assay. The tissue samples were weighed, and their radioactivities were determined using a gamma counter (1480 Wizard 3, Wallace Co., Ltd., Turku, Finland). ^18^F-FMISO-derived radioactivity in the tumors was expressed as the percentage of injected dose per gram of tissue (%ID/g).

### ARG with ^18^F-FMISO

Each excised tumor tissue with calf muscle was sectioned at 2–3 mm thickness to maximize the division surface, embedded in Tissue-Tek medium (Sakura Finetek Europe B.V., Flemingweg, Netherlands), and frozen in isopentane/dry ice. Adjacent cryosections were used for ARG (10 μm) and histological studies (5 μm). The distribution of radioactivity in the tumor tissue was determined by ARG. Briefly, the cryosections were exposed to a phosphor imaging plate (Fuji Imaging Plate BAS-SR 2025 for ^18^F-radioactivity; Fuji Photo Film Co., Ltd., Tokyo, Japan) with a set of calibrated standards [12]. This autoradiographic exposure was performed overnight to detect the distribution of ^18^F-FMISO-derived radioactivity. ARG images were analyzed using a computerized imaging analysis system (FLA-7000 Bio-Imaging Analyzer; Fuji Photo Film Co., Ltd.) with the image analysis software, Multi Gauge (version 3.0; Fuji Photo Film Co., Ltd.). To quantitatively evaluate ^18^F-radioactivity, regions of interest (ROIs) were placed to cover the entire tumor and muscle tissues, excluding the necrotic areas on each ARG image, with reference to the corresponding hematoxylin and eosin (H&E)-stained tissue section. The radioactivity in each ROI was expressed as the percent injected dose per cubic meter of tissue (%ID/cm^3^) determined from photostimulated luminescence per unit area, PSL/mm^2^ (PSL=*a* × *D* × *t*: *a* = constant, *D* = radioactivity exposed on the imaging plate; *t* = exposure time), and the tissue thickness (10 μm).

### Immunohistochemistry

Cryosections (5 μm thick) were immunohistochemically stained for pimonidazole and CD31 to assess hypoxia and microvessel density, respectively. Following rehydration and antigen retrieval, endogenous peroxidase activity was blocked for 10 min in 0.1% methanol supplemented with 0.3% hydrogen peroxide. To assess hypoxia, tissue sections were stained with a rabbit polyclonal anti-pimonidazole antibody (1:200 dilution, Hypoxyprobe, Inc., Burlington, MA, USA) and a Hypoxyprobe-1 Omni kit (Hypoxyprobe, Inc., Burlington, MA, USA). To evaluate the microvessel remodeling, tissue sections were stained using a rabbit polyclonal antibody against CD31 (an endothelial cell marker, 1:50 dilution; Abcam, Cambridge, UK). H&E staining was also performed to assess tumor necrosis.

For the quantitative analysis of hypoxia, the percentage of pimonidazole positive cells in an entire cross-section was determined using ImageJ (Java version 1.6.0; National Institutes of Health, Bethesda, MD, USA). For the quantitative analysis of microvessel density, intratumoral CD31-positive microvessels were counted under an optical microscope (objective magnification, × 400; 0.644 mm^2^ per field), excluding the peripheral connective tissue and central necrotic tissue, with the analyst blind to the purpose of the present study. Single CD31-positive endothelial cells without any visible lumen were not included in the evaluation. A total of > 10 fields per section were randomly analyzed and mean vessel density (MVD, vessels/mm^2^) was determined.

### ^18^F-FMISO PET/CT studies

Mice in the PET imaging study group were divided into eribulin treatment and non-treatment groups (Fig. 1). The mice were anesthetized with 1.0–1.5% isoflurane and injected with 18.5 MBq of ^18^F-FMISO into the tail vein. The mice were placed on a heating sheet in a small animal PET/CT scanner (Inveon; Siemens Medical Solutions USA Inc., Knoxville, TN, USA) in a supine position 4 h after the injection of ^18^F-FMISO. PET and CT were carried out for 10 and 15 min to capture images, respectively. Anesthesia was maintained with 1.0–1.5% isoflurane. After PET/CT, the mice were intraperitoneally treated with a single dose of eribulin (0.3 mg/kg, *n* = 4) or saline (control, *n* = 3) on day 1. Seven days later (day 8), the mice were injected with ^18^F-FMISO and imaged again by PET/CT 4 h after the injection of ^18^F-FMISO following similar procedures described above. The body weight and tumor size were measured on days 1 and 8. The images were reconstructed and corrected for attenuation and scatter using the Fourier rebinning algorithm and filtered back projection with the ramp filter cut-off at the Nyquist frequency. The image matrix was 128 × 128 × 159, resulting in a voxel size of 0.776 × 0.776 × 0.796 mm^3^. The spatial resolution of reconstructed images was 1.63 mm at full-width at half-maximum [22]. Images were analyzed using the Inveon Research Workplace 4.2. A three-dimensional ROI was manually defined for the tumor in each mouse using the PET images with a threshold of one-half of the maximum standardized uptake value (SUV_max_). SUV_max_ was calculated using the single maximum pixel count within the ROI and normalized to the injected dose and mice body weight. Then, the ^18^F-FMISO accumulation level in the tumor was quantified by calculating the mean standardized uptake value (SUV_mean_).

### Statistical analysis

**Fig. 2:**
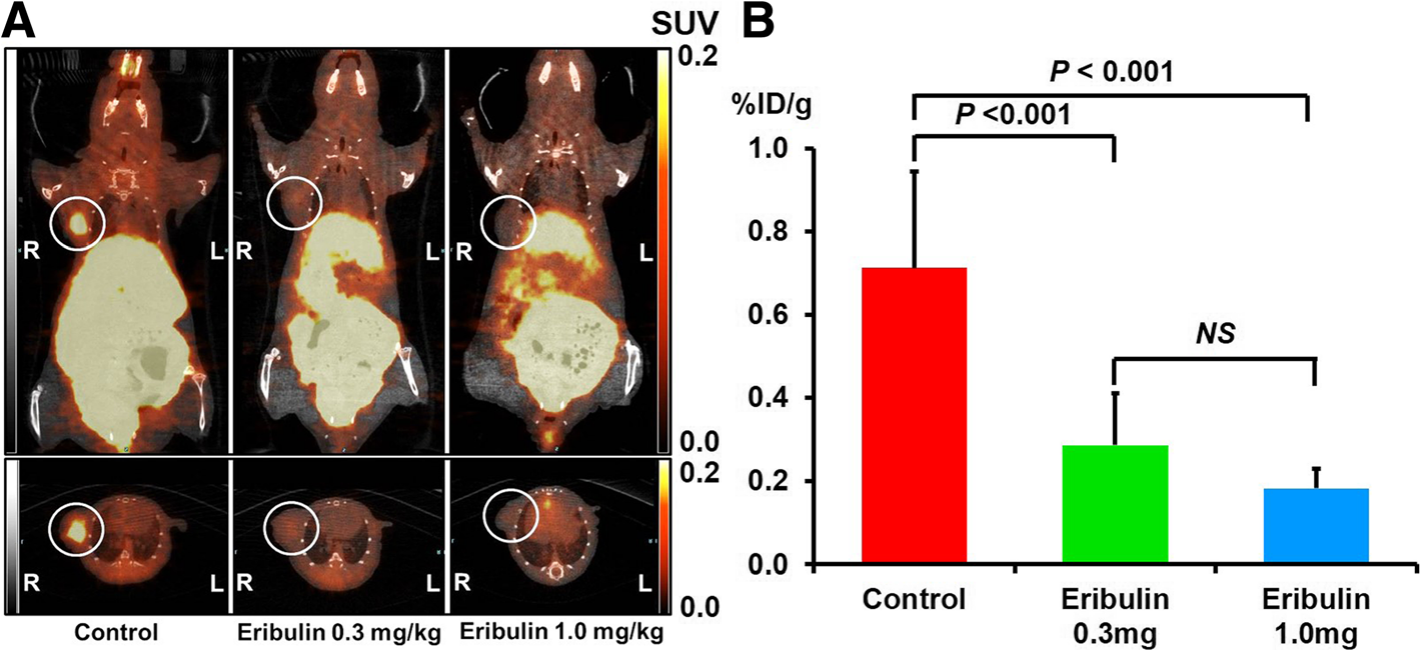
Representative images of ^18^F-FMISO PET (**a**) and intratumoral accumulation levels of ^18^F-FMISO (**b**) in ex vivo study groups. The circle shows the tumor region. R, right; L, left

**Fig. 3:**
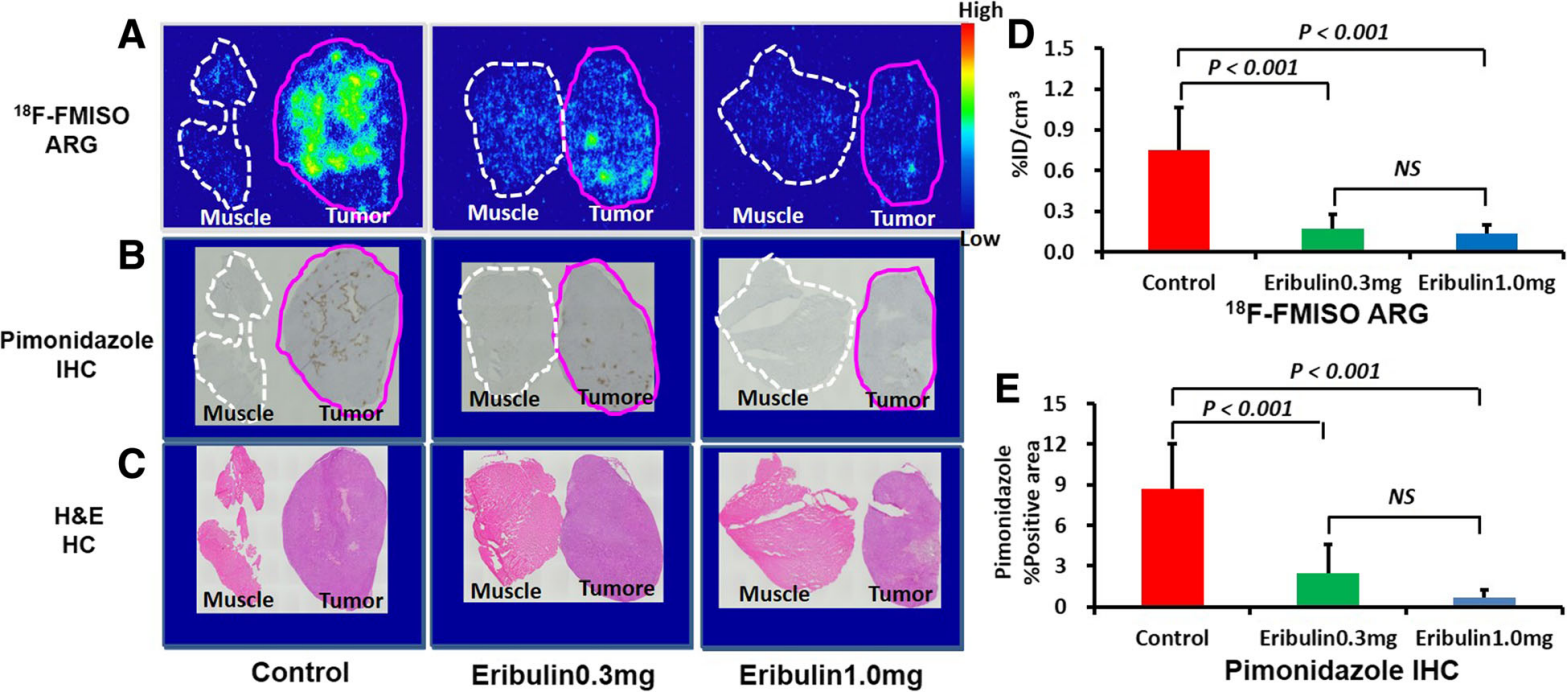
Representative images of ^18^F-FMISO ARG (**a**), pimonidazole IHC (**b**), H&E staining (**c**), quantitative analysis of intratumoral ^18^F-FMISO accumulation level (**d**), and %pimonidazole-positive area (**e**) in the ex vivo study groups

**Fig. 4:**
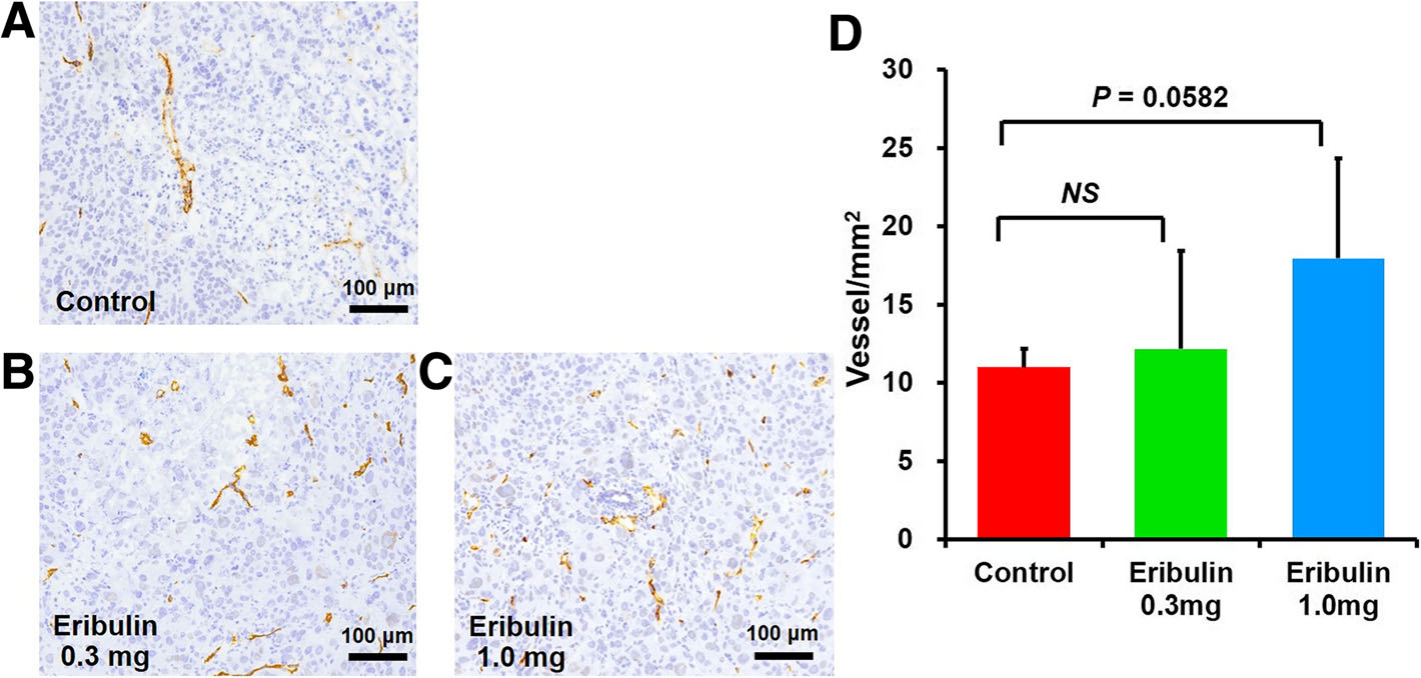
Representative images (**a**–**c**) and quantitative analysis (**d**) of CD31 IHC in the ex vivo groups. Scale bar is 100 μm

**Fig. 5:**
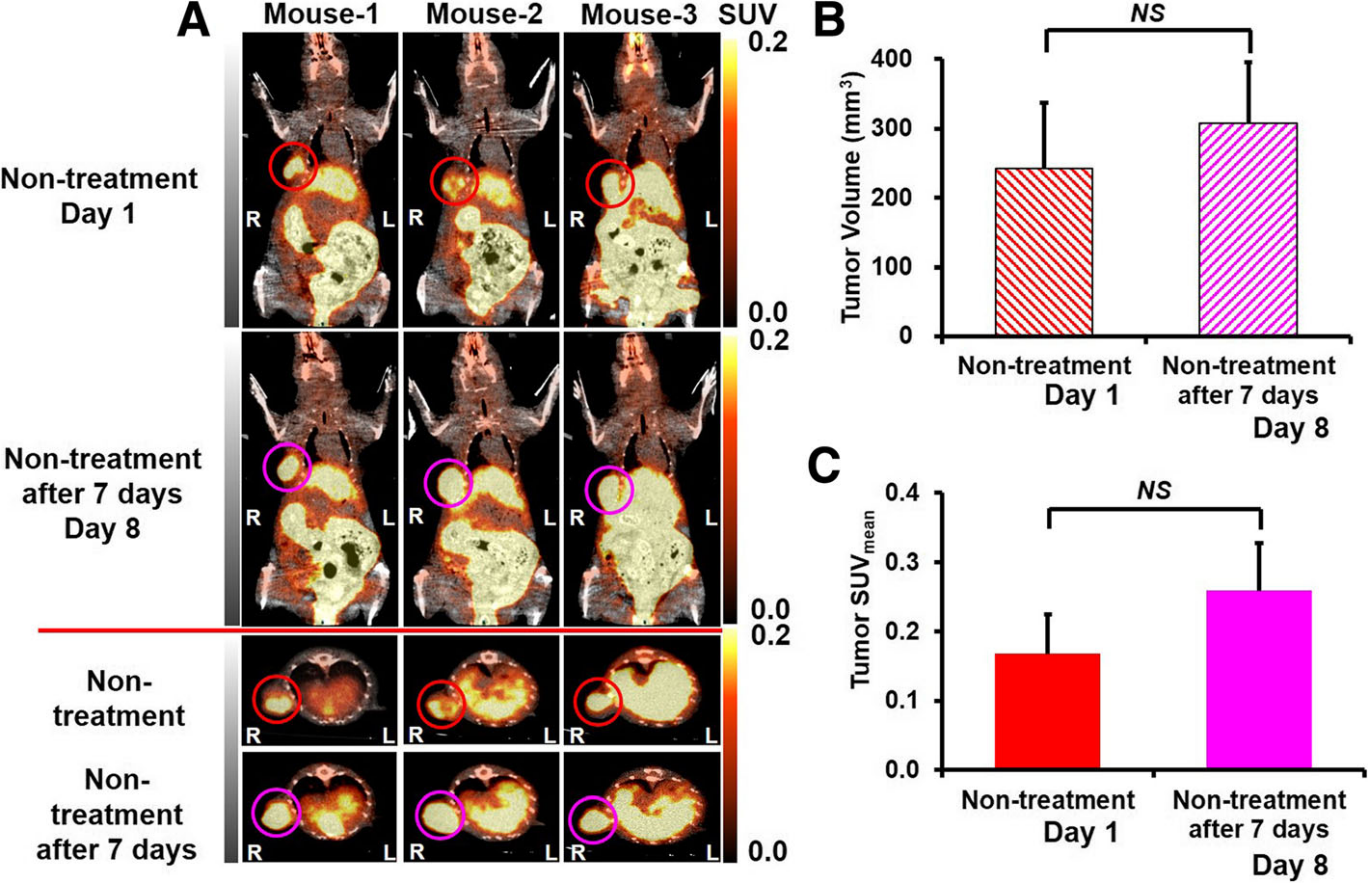
^18^F-FMISO PET images (**a**), tumor volumes (**b**), and quantitative analyses of tumor mean SUV (SUV_mean_) (**c**) in control mice of PET imaging study group

**Fig. 6:**
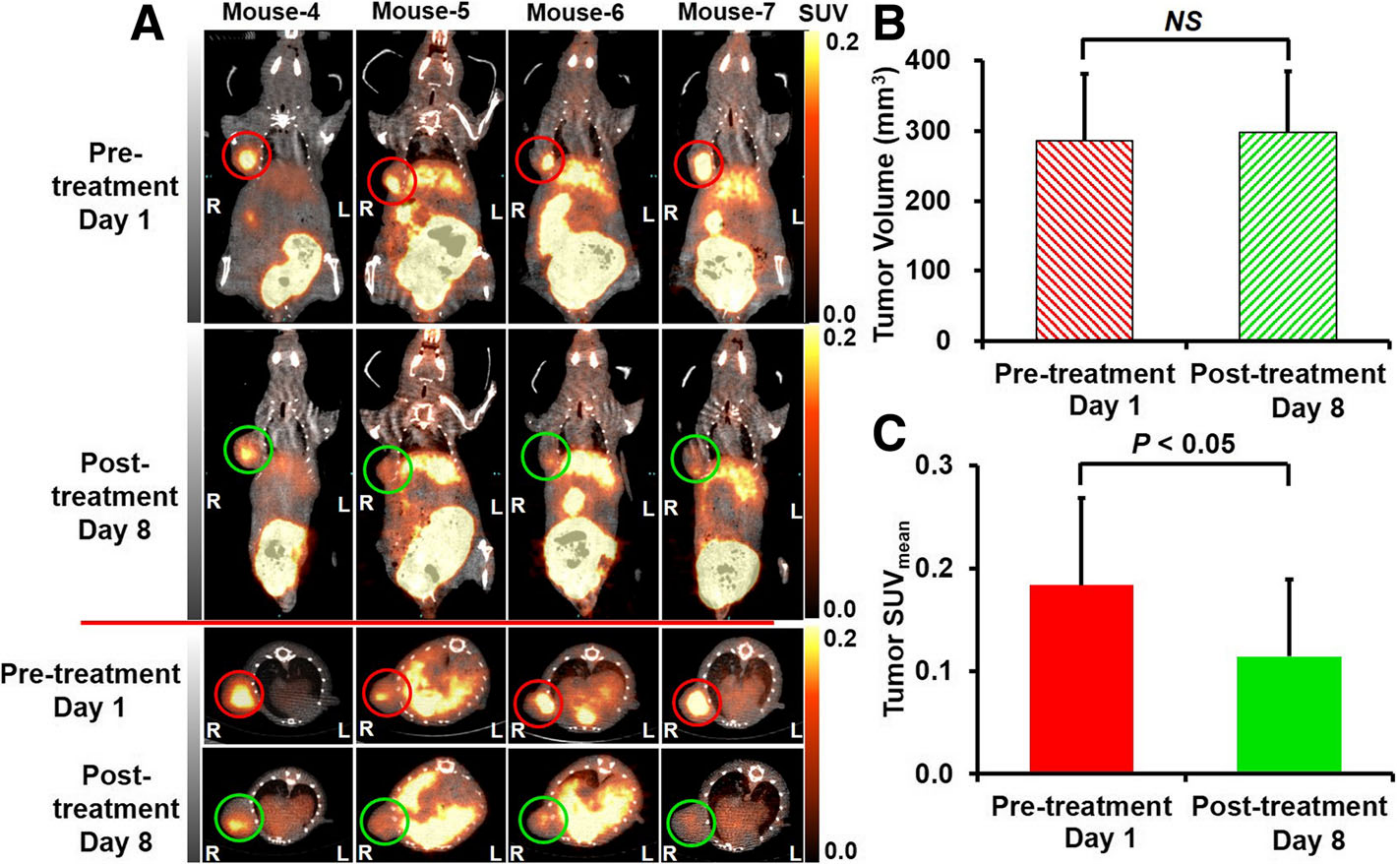
^18^F-FMISO PET images (**a**), tumor volumes (**b**), and quantitative analyses of tumor mean SUV (SUV_mean_) (**c**) in mice treated with eribulin in PET imaging study group

All numeric data are expressed as mean ± standard deviation. In the comparison among three groups (Figs. 2, 3, and 4), one-way analysis of variance (ANOVA) followed by a Bonferroni post hoc test was performed to assess the significance of differences. A paired *t* test was performed to compare the differences in body weight, tumor volume, and SUV_mean_ between the measurement days in the same animal (Tables 1 and 2, Figs. 5 and 6). Significance was assumed at *P* < 0.05.

**Table 1:** Mouse body weight (g)

	Ex vivo study groups (*n* = 16)		PET imaging study groups (*n* = 7)			
			Non-treatment (*n* = 3)		Eribulin treatment (*n* = 4)	
	Day 1	Day 4	Day 1	Day 8	Day 1	Day 8
Control	21.3 ± 1.0	21.8 ± 1.1	23.1 ± 1.2	22.8 ± 1.3	–	–
Eribulin 0.3 mg/kg	20.6 ± 0.4	21.2 ± 0.8	–	–	22.6 ± 1.0	21.1 ± 1.4
Eribulin 1.0 mg/kg	20.1 ± 0.9	20.5 ± 0.7	–	–	–	–

**Table 2:** Tumor volume (mm^3^)

	Ex vivo study groups (*n* = 16)		PET imaging study groups (*n* = 7)			
			Non-treatment (*n* = 3)		Eribulin treatment (*n* = 4)	
	Day 1	Day 4	Day 1	Day 8	Day 1	Day 8
Control	263.0 ± 1.0	290.2 ± 34.2	241.8 ± 95.6	307.2 ± 87.3	–	–
Eribulin 0.3 mg/kg	258.8 ± 40.8	247.5 ± 39.1	–	–	285.6 ± 101.8	297.6.1 ± 113.8
Eribulin 1.0 mg/kg	250.2 ± 35.0	234.4 ± 42.9	–	–	–	–

## Results

### Ex vivo study

#### Mouse body weight and tumor volumes

No significant changes in the body weight (Table 1) and tumor volume (Table 2) were observed between days 1 and 4 in all ex vivo study groups.

#### Visual analysis of ^18^F-FMISO PET, and tissue counting assay and ARG of ^18^F-FMISO

^18^F-FMISO distribution in the tumor was clearly visualized by PET in the control group. The ^18^F-FMISO accumulation level in the tumor was markedly lower in both mouse groups treated with 0.3 and 1.0 mg/kg eribulin than in the control mice (Fig. 2a). In tissue counting assay, ^18^F-FMISO accumulation levels in tumor tissues were significantly lower in mice treated with 0.3 and 1.0 mg/kg eribulin: 38% (0.27 ± 0.12%ID/g) and 26% (0.18 ± 0.05%ID/g) of the control value (0.71 ± 0.23%ID/g), respectively (Fig. 2b).

^18^F-FMISO distribution in the tumor was clearly visualized by ARG in the control group. ^18^F-FMISO distribution was markedly lower in the tumors of both the eribulin-treated groups (Fig. 3a upper figures). Quantitative analysis of ARG images also showed that ^18^F-FMISO accumulation levels in tumor tissues were significantly lower in mice treated with 0.3 and 1.0 mg/kg eribulin: 23% (0.17 ± 0.10%ID/cm^3^) and 18% (0.13 ± 0.06%ID/cm^3^) of the control value (0.75 ± 0.31%ID/cm^3^), respectively (Fig. 3d).

### Histological staining

Compared with the control group, the percent pimonidazole-positive areas were also significantly lower in the treatment groups: 28% (2.5 ± 2.1% pimonidazole-positive area) and 8% (0.7 ± 0.6% pimonidazole-positive area) of the control value (8.7 ± 3.3% pimonidazole-positive area), respectively, for 0.3 and 1.0 mg/kg eribulin treatments (Figs. 3b, e). Immunohistochemistry of CD31 showed that the number of microvessels around tumor hypoxic areas was higher in the eribulin-treated groups than in the control group: 1.1-fold (12.1 ± 6.3 vessels/mm^2^) and 1.6-fold (18.0 ± 6.4 vessels/mm^2^) of the control value (11.0 ± 1.2 vessels/mm^2^), respectively, for 0.3 and 1.0 mg/kg eribulin treatments (Fig. 4).

### PET imaging study

#### Mouse body weight and tumor volumes

No significant changes in body weight (Table 1) were observed between days 1 and 8 in the PET-imaging study groups.

In the non-eribulin-treated group, the tumor volume tended to increase, although the change did not reach a significant level between days 1 and 8 (Table 2, and Fig. 5b). No significant changes in tumor volume were observed between pre- (day 1) and post-treatments (day 8) in the eribulin-treated group (Table 2 and Fig. 6b).

#### Analysis of ^18^F-FMISO PET images

In the non-eribulin-treated group, the tumor SUV_mean_ tended to increase, although the change did not reach a significant level between days 1 and 8 (Fig. 5a, c).

In the eribulin-treated group, ^18^F-FMISO distribution in the tumor was clearly visualized by pretreatment PET on day 1 and its extent markedly decreased on day 8, post-treatment with 0.3 mg/kg eribulin (Fig. 6a). The tumor SUV_mean_ also significantly decreased to 41% of the pre-treatment value [0.21 ± 0.11 and 0.12 ± 0.08 for pre- and post-treatments, respectively] (Fig. 6a, c).

## Discussion

Our ex vivo, autoradiographic, and histological studies of the human tumor xenograft models (MDA-MB435s), demonstrated that the ^18^F-FMISO accumulation level in the tumor decreased after eribulin treatment, which was consistent with the decrease in the pimonidazole-positive area and the increase in the number of microvessels. The ^18^F-FMISO PET study further supported the ex vivo results. Thus, by using the hypoxia imaging technique with ^18^F-FMISO, we demonstrated the elimination of the tumor hypoxic condition by eribulin treatment, strongly indicating the anti-hypoxia capability of eribulin based on its antivascular activity and remodeling of the tumor vasculature. Thus, with its high sensitivity, ^18^F-FMISO PET/CT may be a promising modality to evaluate the efficacy of eribulin to improve the tumor hypoxic condition. Ueda et al. demonstrated that breast tumors had high concentrations of oxyhemoglobin (O_2_Hb) and deoxyhemoglobin (HHb) and low oxygen saturation (SO_2_) level, whereas the tumors showed a decrease in HHb concentration and an increase in SO_2_ level after eribulin treatment [23]. A decrease in tumor HHb concentration may reflect processes counteracting elevated interstitial fluid pressure caused by high vessel permeability, low lymphatic drainage, and poor perfusion. Increased tumor SO_2_ could indicate increased perfusion and reoxygenation. That is, eribulin improves venous efflux, regains tumor perfusion, and enhances oxygenation regardless of therapeutic response. In contrast to eribulin, bevacizumab, a humanized anti-VEGF monoclonal antibody, is known to play a pivotal role in inhibiting angiogenesis and to improve tumor perfusion which significantly decreased tumor O_2_Hb concentrations. That is, bevacizumab inhibits arterial influx and induces hypoxia if vessel remodeling does not work properly. In the presence of vessel remodeling, bevacizumab would improve oxygenation. This result indicated that the mechanism of action of these two agents differs [23]. This result indicated that eribulin treatment could increase perfusion and induce tumor reoxygenation.

Hypoxia, arising from oxygen diffusion limitations, is a hallmark in the pathogenesis of tumor progression and angiogenesis, and is associated with cancer resistance towards radiotherapy and chemotherapy [24–26]. In healthy tissues, oxygen insufficiency often induces cell death. However, in tumors, gradual hypoxia can make tumor cells adapt to this condition by upregulating the production of numerous proteins that promote their survival, changing gene expression to suppress apoptosis, enhancing epithelial-mesenchymal transition, switching anabolism, enhancing the generation of reactive oxygen species (ROS), and downregulating DNA repair pathways [18, 26]. These changes result in poor outcome of treatment in patients. Finding a way to reoxygenate a tumor hypoxic environment may be a key for antitumor treatment. Accordingly, the present results indicate that PET imaging using ^18^F-FMISO may provide the possibility to detect the early treatment response in clinical patients undergoing eribulin treatment.

In recent years, on the basis of the antivascular activity of eribulin and the remodeling of tumor vasculature, several studies have been carried out to clarify the elimination of tumor hypoxic condition by eribulin treatment. Funahashi et al. found that eribulin induces remodeling of the tumor vasculature through its novel antivascular activity, which enhanced the perfusion and vascular function after eribulin treatment using a dynamic contrast-enhanced (DCE)-MRI analyses in the human breast cancer xenograft models [10]. However, their study did not have adequate imaging evidence except an indirect hypoxia assessment by measuring tumor perfusion. Ueda et al. indicated that eribulin treatment can suppress the aggregation of intertumoral deoxyhemoglobin (HHb) and enhance oxygen saturation (SO_2_) by diffuse optical spectroscopic imaging (DOSI) [23]. Although DOSI reflects the hypoxic condition, its low image resolution with difficult quantification would limit its application prospects. In this study, using the hypoxia imaging technique with ^18^F-FMISO, we directly demonstrated the elimination of the tumor hypoxic condition by eribulin treatment. On the other hand, antiangiogenic agents can increase tumor hypoxia by inhibition of angiogenesis. For example, bevacizumab is known to induce a transient phase of vascular normalization, which may enhance drug delivery [27]. However, if the existing vasculature is destroyed without subsequent reorganization, bevacizumab could lead to further hypoxia [23]. Our previous studies confirmed that sorafenib, an antiangiogenic agent, leads to tumor starvation and induced tumor hypoxia in the renal cell carcinoma xenograft. In these studies, the ^18^F-FMISO accumulation level in the tumor increased sorafenib-dose-dependently, which is consistent with the increase in the number of pimonidazole-positive cells and the decrease in the number of microvessels. These findings indicated that ^18^F-FMISO hypoxia imaging confirmed the tumor starvation [12, 19]. It is possible that low ^18^F-MISO uptake may also be due to reduced perfusion and decreased delivery of ^18^F-MISO. However, Funahashi et al. confirmed by DCE-MRI that perfusion of breast tumor cores increased after eribulin treatment [10]. ^18^F-FMISO PET/CT enabled us to non-invasively and quantitatively image hypoxic tumors with a high sensitivity, which are highly advantageous for clinical applications.

In both animal and clinical studies, recent evidence indicates that eribulin can induce tumor vasculature remodeling [10, 23, 28]. In this study, we found that eribulin could alleviate tumor hypoxia, concordant with the increase in the number of CD31^+^ microvessels. These findings would suggest that new blood vessels quickly formed around the tumor hypoxic region and improved the hypoxic environment under the influence of eribulin.

Our results also showed that there is a tendency for the tumor volume to increase in non-treated mice, whereas the tumor progression was suppressed by the eribulin treatment. These findings suggest that eribulin has a powerful capability to inhibit tumor proliferation. The eribulin-induced mitotic arrest is based on the suppression of microtubule growth rate, length, and duration caused by the direct binding of eribulin to microtubule ends with unliganded soluble tubulin [8]. Under the sequestration of tubulin into unusual aggregates, abnormal mitotic spindles cannot pass the metaphase/anaphase checkpoint, and they form an irreversible mitotic blockage, which subsequently leads to cell death by apoptosis [29, 30]. The characteristics of eribulin may explain its capability to inhibit tumor proliferation.

Several studies show that eribulin can render residual tumors less aggressive and less metastatic by reversing EMT to MET states [9, 31]. EMT has been found to play important roles in tumor progression by decreasing intercellular cohesion, increasing cellular migration, and advancing resistance to anticancer agents [32, 33]. Eribulin treatment can significantly suppress the signal pathway of transforming growth factor (TGF)-β, vascular endothelial growth factor (VEGF), and basic fibroblast growth factor (bFGF) after the start of drug infusion [23, 34–36]. With this suppressive property, eribulin can inhibit neoangiogenesis, reverse EMT, and induce MET in tumor tissue [9, 31, 32]. Multiple gene expression analyses have identified gene signatures associated with TGF-β1 signaling which are linked to the acquisition of EMT and stem-cell-like phenotypes exhibited by breast cancer cells [37]. Yoshida et al. reported that eribulin monotherapy of triple-negative breast cancer cells significantly upregulated the mRNA expression of epithelial markers and simultaneously decreased the levels of several mesenchymal markers, leading to the inhibition of neoangiogenesis and the reversal of EMT in a TGF-β1-induced EMT model [9]. Therefore, on the basis of both the present and previous findings, we considered that eribulin might be an efficient therapeutic agent for eliminating tumor hypoxic condition, although the mechanism by which eribulin treatment eliminates the tumor hypoxic condition has not yet been completely elucidated.

The limitation of our study was that only one tumor xenograft model (MDA-MB-435S tumor xenograft) was used to evaluate the effects of eribulin on the tumor hypoxic condition using ^18^F-FMISO hypoxia imaging. MDA-MB-435S cells have been widely used as a highly metastatic human tumor xenograft model for in vivo animal studies. The tumor xenograft was associated with significantly increased hypoxia and reduced necrosis and was used to measure tumor tissue perfusion using ^18^F-FMISO PET imaging [38]. Also, preclinical antitumor activity of eribulin has been widely investigated in this xenograft model [39]. However, it is known that there are inherent intra- and intertumoral variations in tumor hypoxia. Therefore, we consider that various human xenografts, including breast cancer, soft tissue sarcoma, and non-small cell lung cancer, etc., should be used to further confirm our present results.

## Conclusions

In conclusion, by using a hypoxia imaging technique with ^18^F-FMISO, we demonstrated the elimination of the tumor hypoxic condition by eribulin treatment, indicating the antivascular activity of eribulin and remodeling of the tumor vasculature. These findings indicate that PET imaging using ^18^F-FMISO may provide the possibility to detect the early treatment response in clinical patients undergoing eribulin treatment.

## Data Availability

Please contact the corresponding author for data request.

## Abbreviations

^18^F-FMISO: ^18^F-Fluoromisonidazole
PET: Positron emission tomography
PET/CT: PET/computed tomography
FDA: Food and Drug Administration
EMT: Epithelial-mesenchymal transition
MET: Mesenchymal-epithelial transition
pO_2_: Oxygen partial pressure
AAALAC: Association for Assessment and Accreditation of Laboratory Animal Care
ARG: Autoradiography
%ID/g: Percentage of injected dose per gram of tissue
ROIs: Regions of interest
IHC: Immunohistochemistry
MVD: Mean vessel density
SUV_max_: Maximum standardized uptake value
SUV_mean_: Mean standardized uptake value
O_2_Hb: Oxyhemoglobin
HHb: Deoxyhemoglobin
SO_2_: Oxygen saturation
ROS: Reactive oxygen species
DCE: Dynamic contrast-enhanced
MRI: Magnetic resonance imaging
DOSI: Diffuse optical spectroscopic imaging
TGF-β: Transforming growth factor
VEGF: Vascular endothelial growth factor
bFGF: Basic fibroblast growth factor
